# Semaphorin 3F and Netrin-1: The Novel Function as a Regulator of Tumor Microenvironment

**DOI:** 10.3389/fphys.2018.01662

**Published:** 2018-11-23

**Authors:** Hironao Nakayama, Chiaki Kusumoto, Masako Nakahara, Akira Fujiwara, Shigeki Higashiyama

**Affiliations:** ^1^Department of Medical Science and Technology, Hiroshima International University, Higashihiroshima, Japan; ^2^Division of Cell Growth and Tumor Regulation, Proteo-Science Center, Ehime University, Toon, Japan

**Keywords:** semaphorin, netrin, cancer, angiogenesis, metastasis, intracellular signaling

## Abstract

Axon guidance molecules play an important role in regulating proper neuronal networking during neuronal development. They also have non-neuronal properties, which include angiogenesis, inflammation, and tumor development. Semaphorin 3F (SEMA3F), a member of the class 3 semaphorins, was initially identified as an axon guidance factor, that repels axons and collapses growth cones. However, SEMA3F has similar effects on endothelial cells (ECs) and tumor cells. In this review, we discuss the novel molecular mechanisms underlying SEMA3F activity in vascular and tumor biology. Recent evidence suggests that SEMA3F functions as a PI3K-Akt-mTOR inhibitor in mammalian cells, including T cells, ECs, and tumor cells. Therefore, SEMA3F may have broad therapeutic implications. We also discuss the key role of axon guidance molecules as regulators of the tumor microenvironment. Netrin-1, a chemoattractant factor in the neuronal system, promotes tumor progression by enhancing angiogenesis and metastasis. Moreover, our recent studies demonstrate that netrin-1/neogenin interactions augment CD4+ T cell chemokinesis and elicit pro-inflammatory responses, suggesting that netrin-1 plays a key role in modulating the function of a tumor and its surrounding cells in the tumor microenvironment. Overall, this review focuses on SEMA3F and netrin-1 signaling mechanisms to understand the diverse biological functions of axon guidance molecules.

## Introduction

The neuronal network is established precisely during development for normal functioning of the neuronal system. To form synaptic connections, a neuron sends out an axon, projecting toward a distant target, and several dendrites to receive signals from the axon terminals of upstream neurons. Throughout the trajectory toward the target growth cone, a specialized structure at the tip of the extending axon is controlled by attractive and repulsive cues in its surroundings, called axon guidance molecules. Neuronal guidance molecules include semaphorins, netrins, ephrins, and Slit, and their corresponding receptors include neuropilins, UNC5, or DCC families, Eph, and roundabouts (Robo). However, it has been shown that axon guidance molecules also have non-neuronal functions, particularly in the development of the immune (Kumanogoh and Kikutani, 2013) and vascular systems (Carmeliet and Tessier-Lavigne, 2005; Klagsbrun and Eichmann, 2005). Moreover, these ligands and receptors have pivotal roles in tumor progression, especially in tumor growth and metastasis (Neufeld and Kessler, 2008; Mehlen et al., 2011). In this review, we will summarize our knowledge of the biological and molecular mechanisms of axon guidance, specifically semaphorins and netrins, focussing on signal transduction in various cell types, tumor cells, ECs, and immune cells. The goal of this article is to describe the evidence that links axon guidance molecules as regulators of the tumor microenvironment.

### Semaphorins and Their Receptors

The semaphorins were first described as repulsive cues of axon pathfindings during neuronal development (Luo et al., 1993). In vertebrates, more than 20 members of the semaphoring family have been identified and characterized into five classes (semaphorin 3–7) based on structural properties; class 3 semaphorins (SEMA3s) are secreted proteins, class 4–6 semaphorins are transmembrane proteins, and semaphorin 7A is a membrane-associated protein (Neufeld and Kessler, 2008). Most semaphorins elicit signals into the cells by plexins, a family of nine transmembrane receptors. The plexins are subdivided into four subfamilies: plexin A1-A4, plexin B1-B3, plexin C1 and plexin D1. The cytoplasmic region of plexins contains a split GTPase-activating protein (GAP) domain that binds directly to small intracellular GTPase. Neuropilins are also key receptors of semaphorins, especially SEMA3s. There are two genes, NRP1 and NRP2, with approximately 50% structural homology and functional domain structure. The extracellular domain is divided into three subdomains. The a1a2 and b1b2 are ligand-binding domains, while the c domain is crucial for dimerization, to form NRP homo- and heterodimerization (He and Tessier-Lavigne, 1997; Chen et al., 1998). SEMA3s bind to NRP1 or NRP2 receptors, subsequently forming complexes with the plexin A family and transduce signals into the cells. However, SEMA3E is an exception because it directly binds to plexin D1 without NRPs (Klagsbrun and Shimizu, 2010).

#### Endothelial Cell

Over the last two decades, it has been clear that the interaction of semaphorins and their receptors, regulates many processes beyond axon guidance, including angiogenesis and immune responses (Klagsbrun and Eichmann, 2005; Kumanogoh and Kikutani, 2013). For example, NRP1 was originally found as a receptor of semaphorin III/D, also known as collapsin-1 or semaphorin 3A (SEMA3A), and contributed to accurate axon pathfinding (He and Tessier-Lavigne, 1997). NRP1 is also expressed on ECs. NRP1 acts as a co-receptor to enhance binding of the vascular endothelial growth factor 165 (VEGF165), but not VEGF121, to VEGF receptor-2 (VEGFR2), thereby inducing chemotactic and mitogenic activity (Soker et al., 1998). Conversely, SEMA3A binding to NRP1 inhibits EC migration. VEGF165 and SEMA3A compete with each other in EC migration, collapsing activity, and NRP1 binding (Miao et al., 1999), therefore, SEMA3A inhibits angiogenesis.

The plexin family receptors also play an important role in regulating angiogenesis. For example, plexin D1, a receptor of SEMA3E, is highly expressed on ECs during development (van der Zwaag et al., 2002). Interestingly, plexin D1 expression is induced by VEGF in tip cell at the vascular front. The interaction of SEMA3E and plexin D1 negatively regulates the VEGF-induced delta-like 4-Notch singling pathway, which controls cell fate decisions between tip and stalk cells (Kim et al., 2011). Moreover, in zebrafish models, SEMA3-plexin D1 signaling restricts angiogenic activity by the proper endothelial abundance of soluble flt1, an alternatively spliced form of the VEGF receptor Flt1 as a powerful secreted decoy protein (Zygmunt et al., 2011).

#### Immune Cell

The role of semaphorins and their receptors in the immune system has become clear in recent years (Kumanogoh and Kikutani, 2013). NRP1 is highly expressed on Foxp3+ CD4+ T helper cells called regulatory T (Treg) cells. Several groups have shown that NRP1 is useful to distinguish thymus-derived naturally occurring Tregs (nTregs) from peripherally generated adaptive Treg (iTreg) cells (Weiss et al., 2012; Yadav et al., 2012). The lack of NRP1 on CD4+ T cells result in enhanced immune responses in experimental autoimmune encephalitis (EAE). NRP1-deficient CD4+ T cells display an increased Th-17 phenotype, enhanced proliferation and cytokine production, and impairs Treg cell function (Solomon et al., 2011).

Among the plexin family members, plexin-A1 has been implicated in dendritic cell (DC) function in the immune system. Plexin A1 is highly expressed in mature DCs. Indeed, plexin A1 expression is induced by the MHC class II transactivator (CIITA) (Wong et al., 2003). Plexin A1-deficient mice impair the generation of antigen-specific T cells, indicating that plexin A1 is required for DC-mediated T cell responses. In addition, SEMA3A produced by lymphatic EC regulates DC migration and trafficking from peripheral tissues, draining lymph nodes. SEMA3A-plexin A1 interaction induces cytoskeletal fiber rearrangement and DC morphological changes, which allows cells to pass through narrow gaps. Interestingly, depletion of plexin A1 in mice, result in abnormalities not only in immune responses but also in bone homeostasis. Plexin A1 forms a complex with the triggering receptor expressed on myeloid cells 2 (TREM2) and the adaptor molecule DNAX-activation protein 12 (DAP12) on osteoclasts. This complex mediates osteoclast differentiation through PLCγ signaling (Takegahara et al., 2006). These findings show that plexin A1 is indispensable in both immune and skeletal systems (Takegahara et al., 2006; Kumanogoh and Kikutani, 2013).

#### Tumor Cell

The semaphorins are also involved in pathological situations such as tumor progression (Capparuccia and Tamagnone, 2009; Rehman and Tamagnone, 2013) and tumor immunity (Kumanogoh and Kikutani, 2013). For example, semaphorin 3B (SEMA3B) and 3F (SEMA3F) were initially identified as tumor suppressors in lung cancer, because small cell lung cancer is correlated with a deletion in the short arm of chromosome 3 (3p21) (Roche et al., 1996; Xiang et al., 1996). In addition, SEMA3F expression is downregulated by ZEB-1, an E-box transcription repressor in lung cancer. ZEB-1 directly binds to the promoter region of the SEMA3F gene and suppresses SEMA3F transcription (Clarhaut et al., 2009). Moreover, SEMA3F and its receptor NRP2 are direct target genes of the tumor suppressor p53, indicating that the SEMA3F-NRP2 signaling is involved in a tumor suppressive pathway (Futamura et al., 2007).

The expression of SEMA3F is consistently downregulated in highly metastatic tumor cells including the prostate, bladder, and melanoma cells *in vitro* and *in vivo* (Bielenberg et al., 2004). Conversely, tumor cells expressing SEMA3F, inhibit cell adhesion, migration *in vitro*, and tumor angiogenesis and metastasis *in vivo* (Bielenberg et al., 2004; Kessler et al., 2004). More recently, we reported that SEMA3E and SEMA3F are powerful inhibitors of proliferation, migration, and the sprouting of infantile hemangioma cells (Nakayama et al., 2015b).

Overall, semaphorins are characterized as multiple functional regulators in several physiological and pathological events. Nevertheless, the molecular mechanisms of semaphorin activity in diverse cell types are poorly understood. Here, we will review our recent data with a focus on the intracellular signaling mechanisms of semaphorin 3F (SEMA3F) in tumors and ECs.

### Regulation of Actin Cytoskeleton by SEMA3F

The majority of research on axon guidance signaling pathways has focused on neuronal cells, especially in the rearmament of the cytoskeleton and plasma membrane in growth cones and axons (Jung et al., 2012). SEMA3A/collapsin-1 was first identified as a repulsive cue that collapses growth cones and repels axons in dorsal root ganglia (DRG) (Luo et al., 1993). Soon after, Klagsbrun and co-workers found that NRP1 is expressed on ECs (Soker et al., 1998) and that SEMA3A inhibits the micro vessel outgrowth from rat aortic rings (Miao et al., 1999). Similarly, SEMA3A collapses the endothelial tip cell, the leading edge of capillaries, and retracts lamellipodia structures in ECs.

**FIGURE 1:**
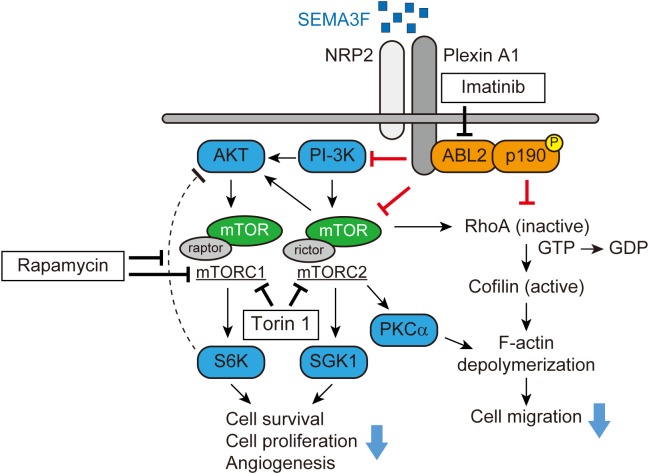
SEMA3F signaling pathways (adapted from Shimizu et al., 2008; Nakayama et al., 2015a). SEMA3F binds to NRP2 and forms a complex with Plexin A1, inactivating PI-3K and mTORC2/Akt-dependent signaling in various cell types. Functionally, these regulatory/pro-resolution signals suppress cell proliferation, migration, cytoskeletal stress fiber rearrangement, and cell survival. SEMA3F also inhibits the cytoskeleton structure in part, by inactivating RhoA through both the ABL2 kinase and p190RhoGAP.

Subsequent studies by the Klagsbrun lab demonstrated that a small GTPase RhoA plays a key role in mediating SEMA3s-induced collapsing activity in tumor cells and ECs (Shimizu et al., 2008; Procaccia et al., 2014). Through phalloidin staining, both SEMA3A and SEMA3F significantly reduce the number of stress fibers in U87MG glioblastoma cells and the human umbilical vein EC (HUVEC). Initially, SEMA3F forms a complex between NRP2 and plexin A1 and recruits ABL2, a membrane-anchored non-receptor tyrosine kinase, and subsequently phosphorylates p190RhoGAP. Activated p190RhoGAP inactivates RhoA, converting GTP to GDP, which phosphorylates cofilin, an actin depolymerizing factor. As a result, active cofilin depolymerizes F-actin, leading to cytoskeletal collapse (Figure 1) (Shimizu et al., 2008). This signaling pathway mediated by SEMA3A and 3F is blocked by Gleevec (imatinib, STI571), an the ABL2 tyrosine kinase inhibitor. Gleevec administration protects tumors and ECs from a SEMA3s-induced cytoskeletal collapse and loss of migration (Procaccia et al., 2014).

### SEMA3F as a Novel mTOR Inhibitor

We profiled levels of phosphokinases in NRP2-expressing tumor cells to determine the inhibitory effect of SEMA3F. In glioblastoma cells, SEMA3F inhibits the phosphorylation of Akt (T308 and S473), Erk, mTOR, and S6K, the mTOR downstream molecule, via NRP2 and plexin A1 receptors (Nakayama et al., 2015a). SEMA3F also inhibits Akt-mTOR signaling in several other cell lines expressing NRP2, including lung cancer cells, melanocytes, Jurkat T lymphocytes, and ECs (Potiron et al., 2007; Nakayama et al., 2015a). Moreover, PI-3K, a regulator of Akt, is also deactivated by SEMA3F in tumor and ECs, suggesting that SEMA3F is a powerfull inhibitor of the PI-3K-Akt-mTOR signaling pathway (Figure 1).

mTOR is a serine/threonine kinase that exists as two distinct multiprotein complexes, composed of either mTOR, raptor, and mLST8 (mTORC1) (Kim et al., 2002; Sancak et al., 2007) or mTOR, rictor, Sin1, protor, and mLST8 (mTORC2) (Sarbassov et al., 2004; Yang et al., 2006; Pearce et al., 2007). mTORC1 and mTORC2 signaling is critical for cell metabolism, differentiation, proliferation, and survival of many normal cell types. Through immunoprecipitation, we found that SEMA3F inhibits the association between mTOR, raptor and rictor in glioblastoma cells, suggesting an inhibitory effect on both mTORC1 and mTORC2, respectively. A recent study showed that invertebrate semaphorin-plexin interactions regulate TOR signaling in *Caenorhabditis elegans* (*C. elegans*), which is required for morphological changes in its epidermal cells (Nukazuka et al., 2011). Rapamycin, an inhibitor of mTOR, markedly inhibits S6K phosphorylation, but induces Akt phosphorylation by blocking the negative feedback loop via mTORC1/S6K (Zhang et al., 2006; Dormond et al., 2007, 2008). Importantly, SEMA3F inhibits both S6K and rapamycin-induced Akt activation when cells were treated with rapamycin and subsequently treated with SEMA3F. This SEMA3F inhibitory effect on Akt-mTOR signaling is similar to that observed in cells treated with the mTORC1/C2 inhibitor Torin 1. SEMA3F also inhibits other known targets of mTORC2, serum/glucocorticoid-regulated kinase-1 (SGK1) and protein kinase C (PKCα), indicating that SEMA3F is a powerful inactivator of mTORC2 (Figure 1) (Nakayama et al., 2015a).

As we previously reported (Shimizu et al., 2008), SEMA3F-NRP2 interactions result in the cytoskeletal collapse in tumor cells and ECs via the RhoA pathway. Since mTORC2 is reported to mediate F-actin cytoskeleton re-organization by interacting with the Rho GTPase family (Sarbassov et al., 2004; Junttila et al., 2009), we tested whether the inhibition of mTORC2 alters F-actin formation in tumor cells. We found that treatment with Torin 1 inhibits the stress fibers in the cells, whereas the mTORC1 inhibitor rapamycin fails to elicit any cytoskeletal changes. Knockdown of rictor (mTORC2) by its specific siRNA, but not raptor (mTORC1), attenuates RhoA activity. Collectively, SEMA3F inactivates RhoA in part via mTORC2 and in part via another mechanism such as the ABL2/p190RhoGAP pathway (Figure 1) (Shimizu et al., 2008; Nakayama et al., 2015a).

We further analyzed the effect of SEMA3F on the regulation of VEGF, a key mediator of angiogenesis. SEMA3F inhibits VEGF promoter activity and secretion induced by the treatment with a hypoxia mimetic agent desferrioxamine (DFO) or hypoxia (1% O_2_) in glioblastoma cells. On the other hand, SEMA3F fails to attenuate VEGF promoter activity in 2DAkt (constitutively active Akt) transfected cells following treatment with DFO (Nakayama et al., 2015a). In lung cancer cells, consistent with our results, SEMA3F downregulates the hypoxia-inducible factor-1α (HIF-1α) protein and VEGF mRNA levels by blocking the Akt-mTOR signaling pathway (Potiron et al., 2007). These findings indicate that SEMA3F suppresses the angiogenic property of tumor cells by inhibiting the production and secretion of VEGF and interrupting VEGF-NRP binding.

There is great interest in targeting mTOR signaling pathways as a therapeutic approach for several diseases such as autoimmune disease, chronic inflammation and allograft rejection, and as an adjunct to cancer therapy (Saxton and Sabatini, 2017). However, many mTOR inhibitors have potential side effects such as immunosuppression and glucose intolerance. Because SEMA3F suppresses rapamycin-induced Akt activation, the combination of SEMA3F with mTOR inhibitors may provide a clinical benefit in cancer therapy.

### Therapeutic Potential of SEMA3F

Several groups, including ours, have demonstrated that SEMA3F inhibits tumor growth, angiogenesis and metastasis in various tumors, such as melanoma (Bielenberg et al., 2004), osteosarcoma (Liu et al., 2016), ovarian cancer (Joseph et al., 2010), colon cancer (Wu et al., 2011; Rao et al., 2015), and lung cancer (Potiron et al., 2007). The effect of SEMA3F on tumor progression has been examined in a well-established xenograft model: parental tumor cells or cells that are engineered to overexpress SEMA3F are implanted into nude mice. For example, high metastatic melanoma cells were transfected with SEMA3F and implanted into mice skin. As a result, the ensuing SEMA3F-tumor did not metastasize into the lymph nodes or lungs. The primary tumors expressing SEMA3F displayed large areas of apoptosis and diminished vascularity (Bielenberg et al., 2004). Similarly, tumor growth was essentially absent when SEMA3F-producing glioblastoma cells were implanted into mice skin compared to parental cells. Additionally, CD31-positive capillaries within the SEMA3F-tumor were constricted and had no discernable lumens (Nakayama et al., 2015a).

In another approach using an *in vivo* model, we injected glioblastoma cells into the skin of nude mice, and after 2 days, the mice received a single intravenous injection of adenovirus encoding human SEMA3F (Ad-3F) or a control adenovirus (Ad-Cont) (Nakayama et al., 2015a). Tumor growth was inhibited over a 14-day period in mice injected with Ad-3F, compared to Ad-Cont-treated. There was a collapsed phenotype of CD31-positive capillaries within tumors in Ad-3F-treated mice. Moreover, through western blot analysis, it was observed that Akt-mTOR signaling was suppressed in tumors of the Ad-3F mice, compared to the control group. In this model, SEMA3F levels were measurable by ELISA or western blot in the serum and the liver, respectively. These results indicate that SEMA3F administration, either local or systemic, has similar anti-tumor and anti-angiogenic effects, by inhibiting the Akt-mTOR singling pathway (Nakayama et al., 2015a).

A recent study showed that a rationally designed SEMA3A mutant, that elicits NRP1-independent signals via the plexin A4 receptor, is an effective anti-tumor agent. Mutant SEMA3A successfully normalized the vasculature, inhibited tumor growth, metastasis, and improved efficacy of chemotherapy in pancreatic cancer mouse models (Gioelli et al., 2018). Based on this study, mutant SEMA3F may also have a similar potential. Moreover, furin-mediated processing of the C-terminal of SEMA3s (SEMA3-95kD) is required as an anti-angiogenic factor, whereas processing in the semaphorin domain (SEMA3-65kD) results in the reduction of repulsive activity (Adams et al., 1997; Parker et al., 2010). These results suggest that the resistant form of the furin protease, SEMA3F-95kD, may be more effective and stable *in vivo*.

It has been reported that NRP1 mediates the infiltration of the Foxp3+ Treg cell into the tumor and regulates the immunological anti-tumor control in response to tumor-derived VEGF (Hansen et al., 2012). The T cell-specific knockout of NRP1 blocks tumor infiltration of Tregs and restores the anti-tumor effect, eventually inhibiting tumor growth. Since SEMA3s compete with VEGF for NRP binding, treatment with SEMA3s may enhance anti-tumor activity by impairing Treg infiltration induced by VEGF. However, the functions of NRP2 in immune systems are poorly understood (Roy et al., 2017; Schellenburg et al., 2017). Further studies are needed to identify the effect of SEMA3F as an immune modulator.

Overall, SEMA3F is a secreted physiological mTOR inhibitor that functions to inhibit tumor growth, angiogenesis, and metastasis. SEMA3F has therapeutic potential, not only in tumor biology, but also to target immune-related diseases such as allograft rejection.

### Netrins and Their Receptors

Netrins are laminin-like proteins first identified as axonal guidance cues, guiding axons during the neural development of *C. elegans* (Ishii et al., 1992). In vertebrates, the netrin family consists of secreted axon guidance molecules that include netrin-1, netrin-3 (also known as netrin-2 chicken-like), and netrin-4. Two other netrins, netrin-G1 and netrin-G2, are characterized by glycosylphosphatidylinositol (GPI) anchorage to the cells. The floor plate-secreted netrin-1 diffuses and establishes a gradient to attract commissural axons expressing netrin receptors to the midline of the central nervous system during the development. Netrin family activity is mediated by several receptors, including uncoordinated 5A-D (UNC5A-D), deleted in colorectal cancer (DCC), its ortholog neogenin, and the Down syndrome cell adhesion molecule (DSCAM). Netrin receptors, called dependence receptors, play a dual role in regulating multiple cellular responses: they mediate positive signals, such as promoting cell proliferation, migration, and survival, in the presence of netrin-1 ligand, while netrin receptors trigger apoptosis under netrin-1 ligand limited conditions (Llambi et al., 2001).

#### Tumor Cell

Netrin-1 also has a number of non-neuronal functions: regulating angiogenesis (Lu et al., 2004; Park et al., 2004; Wilson et al., 2006; Larrivee et al., 2007), inflammation (Tadagavadi et al., 2010; Boneschansker et al., 2016), and tumor progression (Mehlen et al., 2011). For example, the netrin-1 receptor DCC was identified originally as a tumor suppressor in colon cancer associated with a deletion in chromosome 18q21 (Fearon et al., 1990). Loss of netrin-1 receptor expression or inactivation of these receptors are associated with a poor prognosis in patients with colorectal tumors, glioblastoma, and breast carcinoma (Reyes-Mugica et al., 1997; Austrup et al., 2000). On the other hand, upregulation of netrin-1 is observed in some types of cancers. Recently, netrin-1 has been identified as a stimulator of tumor progression in breast cancer (Fitamant et al., 2008), colorectal cancer (Paradisi et al., 2009), gastric cancer (Yin et al., 2017), and medulloblastoma (Akino et al., 2014).

#### Endothelial Cell

However, in the vascular system, it is still controversial whether netrin-1 is a promoter or an inhibitor of angiogenesis. For example, netrin-1 induces proliferation and migration of EC in wound healing (Park et al., 2004). Additionally, netrins accelerated neovascularization and reversed neuropathy and vasculopathy in murine models of ischemia (Wilson et al., 2006). Conversely, activation of UNC5B receptor by netrin-1 mediates repulsive cues for EC sprouting (Larrivee et al., 2007). Disruption of Unc5B in mice or zebrafish increased vessel branching and abnormal navigation, suggesting a negative role for netrin-1 in vasculogenesis (Lu et al., 2004; Larrivee et al., 2007). These inconsistent results might be explained by the bifunctional nature of netrin receptors, which mediate attraction or repulsion, dependent on differential netrin receptor expression levels.

#### Immune Cell

Netrin-1 is also described as a mediator of the immune response. Netrin-1 is expressed in the vascular endothelium; however, its expression is downregulated during infection *in vivo* (Ly et al., 2005). Netrin-1 receptor UNC5B is expressed in leucocytes, netrin-1 acts as a potent inhibitor of the migration of leukocytes *in vitro* and *in vivo* (Ly et al., 2005). Recently, we examined the expression of netrin receptors on human CD4+ T cells. We found that neogenin, UNC5A and UNC5B are expressed by CD4+ T cells at low levels in the steady state; however, protein levels of these receptors are induced following mitogenic activation (Boneschansker et al., 2016). Using a microfluidic assay (Boneschansker et al., 2014), we found that netrin-1 stimulates bidirectional migration of mitogen-activated CD4+ T cells. Knockdown of neogenin by shRNA attenuates netrin-1-induced migratory activity of CD4+ T cells, indicating that netrin-1 mediates promigratory signals via the neogenin receptor. As mentioned above, the biology of netrin-1 is complex because of its potential to interact with attractive or repulsive receptors. In addition, the effects of netrins are even more complex when considering that there are 3 members of the netrin family: netrin-1, netrin-3, and netrin-4. For example, netrin-4 inhibits angiogenesis via neogenin and UNC5B (Lejmi et al., 2008) and suppresses tumor angiogenesis in colorectal cancer (Eveno et al., 2011).

In a humanized SCID mouse model, local injection of netrin-1 into the skin, enhanced immigration of neogenin-expressing CD3+ T cell. Additionally, neogenin is expressed on CD3+ T cell infiltrates within human cardiac allograft biopsies, with evidence of rejection. These results suggest that netrin-1/neogenin interactions augment the T cell chemokinetic response in the process of T cell immigration into human allografts (Boneschansker et al., 2016).

Other evidence shows that netrin-1 promotes atherosclerosis by inhibiting the emigration of macrophages from plaques via the UNC5B receptor (van Gils et al., 2012). Conversely, targeted deletion of netrin-1 in macrophages, attenuates atherosclerosis in mouse models. Moreover, netrin-1 is highly expressed in adipose tissue in obese mice and promotes macrophage retention via UNC5B, and eventually enhances chronic inflammation and insulin resistance (Ramkhelawon et al., 2014).

Overall, netrin-1 plays a crucial role in regulating acute/chronic inflammation, angiogenesis, and tumor and EC invasiveness. Here, we will discuss the molecular mechanisms of how netrin and its receptors regulate multiple cellular functions, in particular, in tumor cells and ECs.

### Regulation of Actin Cytoskeleton by Netrin-1

Glioblastomas are highly invasive brain tumors. Netrin-1 promotes glioblastoma cell migration and invasion by Transwell coated with gelatin (migration) or Matrigel (invasion) assays. In *in vivo* xenograft models, glioblastoma cells overexpressing netrin-1, display metastatic lesions in lymph nodes after 1-month post-resection of primary tumors implanted subcutaneously on the dorsal flank of nude mice. These results show that netrin-1 stimulates glioblastoma cell invasiveness *in vitro* and *in vivo* (Shimizu et al., 2013). Mechanistically, netrin-1 induces glioblastoma cell stress fiber formation by activating the RhoA and cofilin pathway. This netrin-1 effect is the inverse to that of SEMA3F as mentioned above, which inactivates RhoA, thereby collapsing F-actin cytoskeleton.

Netrin-1 also stimulates EC invasion, sprouting, and tube formation (Shimizu et al., 2013; Akino et al., 2014). Consistent with the results of glioblastoma cells, netrin-1 induces EC stress fiber formation and invasion via a RhoA and cofilin pathway. The neogenin receptor is highly expressed in mouse brain capillary EC. Knockdown of neogenin by siRNA in EC significantly decreases netrin-1-induced EC invasion and tube formation. Moreover, netrin-1 induces CD31-positive EC infiltration into Matrigel, which is abrogated by the neogenin neutralizing antibody.

### Netrin-1 Activates Cathepsin B and CREB

**FIGURE 2:**
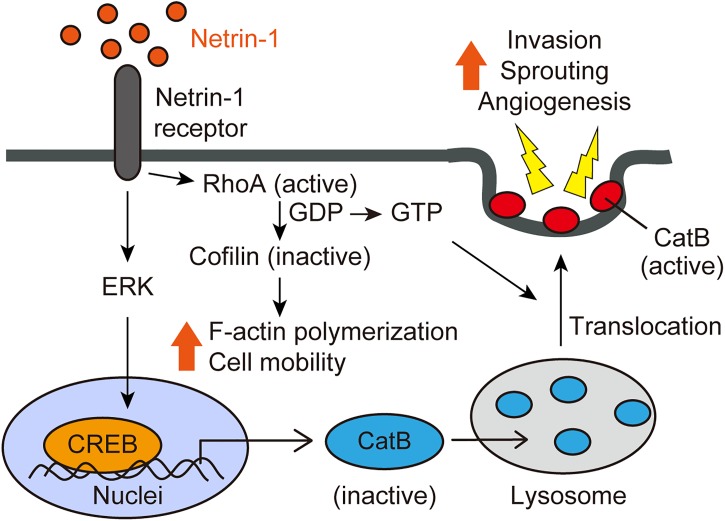
Netrin-1 signaling pathways (adapted from Shimizu et al., 2013). Netrin-1 activates RhoA in glioblastoma cells and ECs, enhancing stress fiber formation and compromising the integrity of the F-actin cytoskeleton. Netrin-1 also induces the phosphorylation of CREB via the MAPK pathway, leading to up-regulation of CatB expression. Netrin-1 promotes the translocation of CatB from the lysosome to the cell surface as an enzymatically active form in a RhoA-dependent manner. Once at the cell surface, CatB acts to promote glioblastoma invasiveness and angiogenesis.

Alternatively, netrin-1-induced cell invasion is mediated by activated cathepsin B (CatB), a cysteine protease that translocates to the cell surface as an active enzyme. The netrin-1-induced translocation of CatB to the cell surface was abrogated by a RhoA inhibitor C3 transferase, indicating that netrin-1-induced CatB activation and translocation were dependent on RhoA activation. The CatB specific inhibitor, CA-074Me, inhibits netrin-1-induced cell invasion. We further investigated the intracellular signaling pathways mediated by netrin-1 with a phosphoprotein antibody array. Netrin-1 induces phosphorylation of the ERK and CREB (cAMP-response element-binding protein) in glioblastoma cells. CREB is a transcription factor involved in the cell invasion and metastasis of tumors (Melnikova et al., 2006; Shi et al., 2011). Knockdown of the CREB reduces glioblastoma cell invasiveness, sprouting and CatB expression. Taken together, netrin-1 activates glioblastoma cell invasion in a RhoA-, CREB-, and CatB-dependent manner (Figure 2) (Shimizu et al., 2013).

### Netrin-1 as a Biomarker of Medulloblastoma

Medulloblastoma is the most common malignant pediatric brain tumor. For patients with invasive or disseminated medulloblastoma, the 5-year survival rate can be as low as 32% (Zeltzer et al., 1999). The mechanisms underlying the invasion and dissemination in medulloblastoma still remain elusive. Based on the results that netrin-1 induces glioblastoma cell invasion (Shimizu et al., 2013), we tested the netrin-1 effect on medulloblastoma. We showed that netrin-1 stimulated medulloblastoma cell invasion via neogenin and UNC5B receptors. Inhibition of netrin-1 by the neutralizing antibody, blocked medulloblastoma cell invasion and reduced phosphorylation of the ERK and CatB expression. Moreover, the MEK inhibitor U0126, reduced CatB expression in medulloblastoma. These inhibition results suggest that netrin-1 secreted by medulloblastoma cells, stimulates medulloblastoma cell invasiveness by activating the CatB via the MAPK pathway (Akino et al., 2014).

In pediatric medulloblastoma patients, netrin-1 mRNA and protein levels are elevated in medulloblastoma specimens compared with control specimens from the same patient. Urinary netrin-1 levels are also higher in medulloblastoma patients compared with controls. Importantly, urinary netrin-1 is higher in patients with invasive medulloblastoma compared with non-invasive medulloblastoma. Notably, urinary netrin-1 levels diminished after the resection of medulloblastoma in patients. These results suggest that the netrin-1-neogenin (or UNC5B) pathway is a promising therapeutic target to inhibit medulloblastoma invasiveness, and that the measurement of netrin-1 might be useful to detect invasive and disseminated phenotypes of medulloblastoma to predict the disease status (Akino et al., 2014).

## Conclusion and Future Perspectives

**FIGURE 3:**
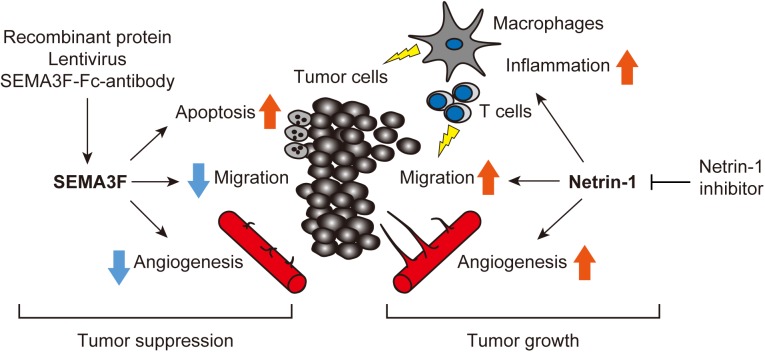
Axon guidance molecules are crucial regulators of the tumor microenvironment. SEMA3F and netrin-1 have multifaceted effects on tumors and surrounding non-tumor cells, including ECs, leukocytes, macrophages, and fibroblasts etc. Treatment with exogenous SEMA3F or the inhibition of netrin-1 signaling would be promising for cancer therapy.

An increasing body of data indicates that axon guidance molecules are crucial regulators of the tumor microenvironment. We have summarized the current evidence that SEMA3F and netrin-1 have multifaceted effects on tumors and surrounding non-tumor cells, including ECs, leukocytes, macrophages, and fibroblasts (Figure 3). Therefore, we propose that targeting SEMA3F and netrin-1 may be a promising strategy for cancer therapy.

As described above, systemic treatment with SEMA3F (full-length or its mutants) is a potential regulator of the tumor microenvironment; however, further studies on the safety and stability of SEMA3F *in vivo* are required for clinical use. On the other hand, regulation of netrin-1 seems to be more complex because it has multiple promotive and suppressive receptors. Therefore, selective inhibition of the interaction of netrin-1 and key receptor(s) involved in tumor progression is essential. To reduce adverse side effects, further studies on the development of inhibitors, such as small molecule compounds or antibodies, to interfere with netrin-1 and its specific receptor are needed.

Axon guidance molecules may regulate the epithelial mesenchymal transition (EMT), playing a critical role in tumor progression, invasion, and metastasis. For example, treatment with sunitinib induces primary tumor shrinkage and inhibits angiogenesis in a mouse model of pancreatic neuroendocrine cancer (RIP-Tag2). However, it concurrently promotes local invasiveness and distant metastasis, indicating that sunitinib activates the EMT program (Maione et al., 2012). Indeed, sunitinib upregulates mesenchymal markers, Snail1, vimentin, and *N*-cadherin in tumors, whereas it inhibits the epithelial marker, *E*-cadherin. Surprisingly, SEMA3A reversed the effects of sunitinib, inhibiting Snail1 and vimentin and inducing *E*-cadherin expression. Further studies are required to understand whether SEMA3F and netrin-1 are involved in the regulation of EMT, especially in the tumor microenvironment.

Inflammation in the tumor microenvironment also affects tumor development and progression as well as the response to therapy (Chen and Nunez, 2011; Karki et al., 2017). A key mechanism driving inflammation in immune cells is induced by the inflammasome, a cytoplasmic multimeric protein complex that mediates activation of caspase-1, which subsequently promotes the secretion of pro-inflammatory cytokines. Among inflammasome complexes, the Nod-like receptor family member NLRP3 is well characterized and contributes to various diseases such as colorectal cancer and melanoma. It has been reported that NLRP3 mediates IL18 secretion to protect individuals from colitis-associated colorectal cancer (Allen et al., 2010; Zaki et al., 2010). It is possible that axon guidance molecules, such as netrin-1, could contribute in the regulation of inflammasomes, because netrin-1 regulates the mobility of immune cells in association with acute/chronic inflammation in pathological conditions.

Finally, we need to understand the function of axon guidance molecules in the maintenance of cancer stem cells (or tumor-initiating cells). Currently, it is not known whether axon guidance molecules modulate cancer stem cell behavior. Given that cancer stem cells play a pivotal role in drug resistance, future studies will provide new approaches to overcome chemotherapy resistance by targeting axon guidance molecules.

## Summary

### SEMA3F Is a Powerful mTOR Inhibitor *in vitro* and *in vivo*

The SEMA3F-NRP2-Plexin A1 interactions inhibit intracellular PI-3K activity, mTORC2-dependent signaling, RhoA activity, and cytoskeletal stress fiber formation. These inhibitory effects are observed in diverse human cell types, suggesting that SEMA3F has broad therapeutic implications.

### Novel Netrin-1 Mechanisms Include Activation of RhoA, CatB, and CREB

Netrin-1 promotes glioblastoma and medulloblastoma cell invasion in a RhoA-dependent manner. CatB activation is crucial for enhancing tumor cell invasiveness and angiogenesis. Targeting mediators such as netrin-1 and CatB could be candidates for brain cancer therapy.

### Axon Guidance Molecules as Biomarker of Tumor

Netrin-1 is a candidate biomarker capable of detecting the invasive, disseminated phenotype in patients with medulloblastoma and predicting their disease status. On the other hand, the expression of SEMA3F is downregulated in highly metastatic tumor cells. Therefore, a balance of netrin-1 and SEMA3F in tumor cells might be a diagnostic and prognostic biomarker for a high metastatic tumor.

### Axon Guidance Molecules Modulate Tumor Microenvironment

SEMA3F and netrin-1 have multifaceted effects on tumor and surrounding non-tumor cells, including ECs, leukocytes, macrophages, and fibroblasts. We propose that targeting SEMA3F and netrin-1 may be a promising strategy for cancer therapy.

## Author Contributions

HN and SH contributed to the design and the concept of the study. HN, CK, and MN contributed to the development of the methodology. HN, CK, MN, and AF acquired the data. HN analyzed and interpreted the data, and contributed to the writing and the revisions of the manuscript.

## Conflict of Interest Statement

The authors declare that the research was conducted in the absence of any commercial or financial relationships that could be construed as a potential conflict of interest.

## References

[B1] Adams R. H. Lohrum M. Klostermann A. Betz H. Puschel A. W. (1997). The chemorepulsive activity of secreted semaphorins is regulated by furin-dependent proteolytic processing. *EMBO J.* 16 6077–6086. 10.1093/emboj/16.20.6077 9321387 PMC1326291

[B2] Akino T. Han X. Nakayama H. McNeish B. Zurakowski D. Mammoto A. (2014). Netrin-1 promotes medulloblastoma cell invasiveness and angiogenesis, and demonstrates elevated expression in tumor tissue and urine of patients with pediatric medulloblastoma. *Cancer Res.* 74 3716–3726. 10.1158/0008-5472.CAN-13-3116 24812271 PMC4123751

[B3] Allen I. C. TeKippe E. M. Woodford R. M. Uronis J. M. Holl E. K. Rogers A. B. (2010). The NLRP3 inflammasome functions as a negative regulator of tumorigenesis during colitis-associated cancer. *J. Exp. Med.* 207 1045–1056. 10.1084/jem.20100050 20385749 PMC2867287

[B4] Austrup F. Uciechowski P. Eder C. Bockmann B. Suchy B. Driesel G. (2000). Prognostic value of genomic alterations in minimal residual cancer cells purified from the blood of breast cancer patients. *Br. J. Cancer* 83 1664–1673. 10.1054/bjoc.2000.1501 11104564 PMC2363462

[B5] Bielenberg D. R. Hida Y. Shimizu A. Kaipainen A. Kreuter M. Kim C. C. (2004). Semaphorin 3F, a chemorepulsant for endothelial cells, induces a poorly vascularized, encapsulated, nonmetastatic tumor phenotype. *J. Clin. Invest.* 114 1260–1271. 10.1172/JCI21378 15520858 PMC524226

[B6] Boneschansker L. Nakayama H. Eisenga M. Wedel J. Klagsbrun M. Irimia D. (2016). Netrin-1 augments chemokinesis in CD4^+^ T cells in vitro and elicits a proinflammatory response in vivo. *J. Immunol.* 197 1389–1398. 10.4049/jimmunol.1502432 27430720 PMC4976028

[B7] Boneschansker L. Yan J. Wong E. Briscoe D. M. Irimia D. (2014). Microfluidic platform for the quantitative analysis of leukocyte migration signatures. *Nat. Commun.* 5:4787. 10.1038/ncomms5787 25183261 PMC4155519

[B8] Capparuccia L. Tamagnone L. (2009). Semaphorin signaling in cancer cells and in cells of the tumor microenvironment–two sides of a coin. *J. Cell Sci.* 122 1723–1736. 10.1242/jcs.030197 19461072

[B9] Carmeliet P. Tessier-Lavigne M. (2005). Common mechanisms of nerve and blood vessel wiring. *Nature* 436 193–200. 10.1038/nature03875 16015319

[B10] Chen G. Y. Nunez G. (2011). Inflammasomes in intestinal inflammation and cancer. *Gastroenterology* 141 1986–1999. 10.1053/j.gastro.2011.10.002 22005480 PMC3442608

[B11] Chen H. He Z. Bagri A. Tessier-Lavigne M. (1998). Semaphorin-neuropilin interactions underlying sympathetic axon responses to class III semaphorins. *Neuron* 21 1283–1290. 10.1016/S0896-6273(00)80648-0 9883722

[B12] Clarhaut J. Gemmill R. M. Potiron V. A. Ait-Si-Ali S. Imbert J. Drabkin H. A. (2009). ZEB-1, a repressor of the semaphorin 3F tumor suppressor gene in lung cancer cells. *Neoplasia* 11 157–166. 10.1593/neo.81074 19177200 PMC2631140

[B13] Dormond O. Contreras A. G. Meijer E. Datta D. Flynn E. Pal S. (2008). CD40-induced signaling in human endothelial cells results in mTORC2- and Akt-dependent expression of vascular endothelial growth factor in vitro and in vivo. *J. Immunol.* 181 8088–8095. 10.4049/jimmunol.181.11.8088 19018001 PMC3495983

[B14] Dormond O. Madsen J. C. Briscoe D. M. (2007). The effects of mTOR-Akt interactions on anti-apoptotic signaling in vascular endothelial cells. *J. Biol. Chem.* 282 23679–23686. 10.1074/jbc.M700563200 17553806 PMC3383050

[B15] Eveno C. Broqueres-You D. Feron J. G. Rampanou A. Tijeras-Raballand A. Ropert S. (2011). Netrin-4 delays colorectal cancer carcinomatosis by inhibiting tumor angiogenesis. *Am. J. Pathol.* 178 1861–1869. 10.1016/j.ajpath.2010.12.019 21406174 PMC3078466

[B16] Fearon E. R. Cho K. R. Nigro J. M. Kern S. E. Simons J. W. Ruppert J. M. (1990). Identification of a chromosome 18q gene that is altered in colorectal cancers. *Science* 247 49–56. 10.1126/science.2294591 2294591

[B17] Fitamant J. Guenebeaud C. Coissieux M. M. Guix C. Treilleux I. Scoazec J. Y. (2008). Netrin-1 expression confers a selective advantage for tumor cell survival in metastatic breast cancer. *Proc. Natl. Acad. Sci. U.S.A.* 105 4850–4855. 10.1073/pnas.0709810105 18353983 PMC2290782

[B18] Futamura M. Kamino H. Miyamoto Y. Kitamura N. Nakamura Y. Ohnishi S. (2007). Possible role of semaphorin 3F, a candidate tumor suppressor gene at 3p21.3, in p53-regulated tumor angiogenesis suppression. *Cancer Res.* 67 1451–1460. 10.1158/0008-5472.CAN-06-2485 17308083

[B19] Gioelli N. Maione F. Camillo C. Ghitti M. Valdembri D. Morello N. (2018). A rationally designed NRP1-independent superagonist SEMA3A mutant is an effective anticancer agent. *Sci. Transl. Med.* 10:eaah4807. 10.1126/scitranslmed.aah4807 29794061

[B20] Hansen W. Hutzler M. Abel S. Alter C. Stockmann C. Kliche S. (2012). Neuropilin 1 deficiency on CD4^+^Foxp3^+^ regulatory T cells impairs mouse melanoma growth. *J. Exp. Med.* 209 2001–2016. 10.1084/jem.20111497 23045606 PMC3478934

[B21] He Z. Tessier-Lavigne M. (1997). Neuropilin is a receptor for the axonal chemorepellent Semaphorin III. *Cell* 90 739–751. 10.1016/S0092-8674(00)80534-6 9288753

[B22] Ishii N. Wadsworth W. G. Stern B. D. Culotti J. G. Hedgecock E. M. (1992). UNC-6, a laminin-related protein, guides cell and pioneer axon migrations in *C. elegans*. *Neuron* 9 873–881. 10.1016/0896-6273(92)90240-E 1329863

[B23] Joseph D. Ho S. M. Syed V. (2010). Hormonal regulation and distinct functions of semaphorin-3B and semaphorin-3F in ovarian cancer. *Mol. Cancer Ther.* 9 499–509. 10.1158/1535-7163.MCT-09-0664 20124444 PMC2820590

[B24] Jung H. Yoon B. C. Holt C. E. (2012). Axonal mRNA localization and local protein synthesis in nervous system assembly, maintenance and repair. *Nat. Rev. Neurosci.* 13 308–324. 10.1038/nrn3210 22498899 PMC3682205

[B25] Junttila T. T. Akita R. W. Parsons K. Fields C. Lewis Phillips G. D. Friedman L. S. (2009). Ligand-independent HER2/HER3/PI3K complex is disrupted by trastuzumab and is effectively inhibited by the PI3K inhibitor GDC-0941. *Cancer Cell* 15 429–440. 10.1016/j.ccr.2009.03.020 19411071

[B26] Karki R. Man S. M. Kanneganti T. D. (2017). Inflammasomes and Cancer. *Cancer Immunol. Res.* 5 94–99. 10.1158/2326-6066.CIR-16-0269 28093447 PMC5593081

[B27] Kessler O. Shraga-Heled N. Lange T. Gutmann-Raviv N. Sabo E. Baruch L. (2004). Semaphorin-3F is an inhibitor of tumor angiogenesis. *Cancer Res.* 64 1008–1015. 10.1158/0008-5472.CAN-03-3090 14871832

[B28] Kim D. H. Sarbassov D. D. Ali S. M. King J. E. Latek R. R. Erdjument-Bromage H. (2002). mTOR interacts with raptor to form a nutrient-sensitive complex that signals to the cell growth machinery. *Cell* 110 163–175. 10.1016/S0092-8674(02)00808-5 12150925

[B29] Kim J. Oh W. J. Gaiano N. Yoshida Y. Gu C. (2011). Semaphorin 3E-Plexin-D1 signaling regulates VEGF function in developmental angiogenesis via a feedback mechanism. *Genes Dev.* 25 1399–1411. 10.1101/gad.2042011 21724832 PMC3134083

[B30] Klagsbrun M. Eichmann A. (2005). A role for axon guidance receptors and ligands in blood vessel development and tumor angiogenesis. *Cytokine Growth Factor Rev.* 16 535–548. 10.1016/j.cytogfr.2005.05.002 15979925

[B31] Klagsbrun M. Shimizu A. (2010). Semaphorin 3E, an exception to the rule. *J. Clin. Invest.* 120 2658–2660. 10.1172/JCI44110 20664165 PMC2912208

[B32] Kumanogoh A. Kikutani H. (2013). Immunological functions of the neuropilins and plexins as receptors for semaphorins. *Nat. Rev. Immunol.* 13 802–814. 10.1038/nri3545 24319778

[B33] Larrivee B. Freitas C. Trombe M. Lv X. Delafarge B. Yuan L. (2007). Activation of the UNC5B receptor by Netrin-1 inhibits sprouting angiogenesis. *Genes Dev.* 21 2433–2447. 10.1101/gad.437807 17908930 PMC1993874

[B34] Lejmi E. Leconte L. Pedron-Mazoyer S. Ropert S. Raoul W. Lavalette S. (2008). Netrin-4 inhibits angiogenesis via binding to neogenin and recruitment of Unc5B. *Proc. Natl. Acad. Sci. U.S.A.* 105 12491–12496. 10.1073/pnas.0804008105 18719102 PMC2518829

[B35] Liu M. H. Fu W. J. Cui Y. H. Guo Q. N. Zhou Y. (2016). Downregulation of Semaphorin-3F is associated with poor prognostic significance in osteosarcoma patients. *Am. J. Cancer Res.* 6 2252–2262. 27822415 PMC5088289

[B36] Llambi F. Causeret F. Bloch-Gallego E. Mehlen P. (2001). Netrin-1 acts as a survival factor via its receptors UNC5H and DCC. *EMBO J.* 20 2715–2722. 10.1093/emboj/20.11.2715 11387206 PMC125255

[B37] Lu X. Le Noble F. Yuan L. Jiang Q. De Lafarge B. Sugiyama D. (2004). The netrin receptor UNC5B mediates guidance events controlling morphogenesis of the vascular system. *Nature* 432 179–186. 10.1038/nature03080 15510105

[B38] Luo Y. Raible D. Raper J. A. (1993). Collapsin: a protein in brain that induces the collapse and paralysis of neuronal growth cones. *Cell* 75 217–227. 10.1016/0092-8674(93)80064-L 8402908

[B39] Ly N. P. Komatsuzaki K. Fraser I. P. Tseng A. A. Prodhan P. Moore K. J. (2005). Netrin-1 inhibits leukocyte migration in vitro and in vivo. *Proc. Natl. Acad. Sci. U.S.A.* 102 14729–14734. 10.1073/pnas.0506233102 16203981 PMC1253572

[B40] Maione F. Capano S. Regano D. Zentilin L. Giacca M. Casanovas O. (2012). Semaphorin 3A overcomes cancer hypoxia and metastatic dissemination induced by antiangiogenic treatment in mice. *J. Clin. Invest.* 122 1832–1848. 10.1172/JCI58976 22484816 PMC3336974

[B41] Mehlen P. Delloye-Bourgeois C. Chedotal A. (2011). Novel roles for Slits and netrins: axon guidance cues as anticancer targets? *Nat. Rev. Cancer* 11 188–197. 10.1038/nrc3005 21326323

[B42] Melnikova V. O. Mourad-Zeidan A. A. Lev D. C. Bar-Eli M. (2006). Platelet-activating factor mediates MMP-2 expression and activation via phosphorylation of cAMP-response element-binding protein and contributes to melanoma metastasis. *J. Biol. Chem.* 281 2911–2922. 10.1074/jbc.M508683200 16306050

[B43] Miao H. Q. Soker S. Feiner L. Alonso J. L. Raper J. A. Klagsbrun M. (1999). Neuropilin-1 mediates collapsin-1/semaphorin III inhibition of endothelial cell motility: functional competition of collapsin-1 and vascular endothelial growth factor-165. *J. Cell Biol.* 146 233–242. 10.1083/jcb.146.1.233 10402473 PMC2199727

[B44] Nakayama H. Bruneau S. Kochupurakkal N. Coma S. Briscoe D. M. Klagsbrun M. (2015a). Regulation of mTOR signaling by semaphorin 3F-neuropilin 2 interactions in vitro and in vivo. *Sci. Rep.* 5:11789. 10.1038/srep11789 26156437 PMC4496725

[B45] Nakayama H. Huang L. Kelly R. P. Oudenaarden C. R. Dagher A. Hofmann N. A. (2015b). Infantile hemangioma-derived stem cells and endothelial cells are inhibited by class 3 semaphorins. *Biochem. Biophys. Res. Commun.* 464 126–132. 10.1016/j.bbrc.2015.06.087 26086095 PMC6549231

[B46] Neufeld G. Kessler O. (2008). The semaphorins: versatile regulators of tumour progression and tumour angiogenesis. *Nat. Rev. Cancer* 8 632–645. 10.1038/nrc2404 18580951

[B47] Nukazuka A. Tamaki S. Matsumoto K. Oda Y. Fujisawa H. Takagi S. (2011). A shift of the TOR adaptor from Rictor towards Raptor by semaphorin in *C. elegans*. *Nat. Commun.* 2:484. 10.1038/ncomms1495 21952218 PMC3195255

[B48] Paradisi A. Maisse C. Coissieux M. M. Gadot N. Lepinasse F. Delloye-Bourgeois C. (2009). Netrin-1 up-regulation in inflammatory bowel diseases is required for colorectal cancer progression. *Proc. Natl. Acad. Sci. U.S.A.* 106 17146–17151. 10.1073/pnas.0901767106 19721007 PMC2761333

[B49] Park K. W. Crouse D. Lee M. Karnik S. K. Sorensen L. K. Murphy K. J. (2004). The axonal attractant Netrin-1 is an angiogenic factor. *Proc. Natl. Acad. Sci. U.S.A.* 101 16210–16215. 10.1073/pnas.0405984101 15520390 PMC528958

[B50] Parker M. W. Hellman L. M. Xu P. Fried M. G. Vander Kooi C. W. (2010). Furin processing of semaphorin 3F determines its anti-angiogenic activity by regulating direct binding and competition for neuropilin. *Biochemistry* 49 4068–4075. 10.1021/bi100327r 20387901 PMC2868107

[B51] Pearce L. R. Huang X. Boudeau J. Pawlowski R. Wullschleger S. Deak M. (2007). Identification of Protor as a novel Rictor-binding component of mTOR complex-2. *Biochem. J.* 405 513–522. 10.1042/BJ20070540 17461779 PMC2267312

[B52] Potiron V. A. Sharma G. Nasarre P. Clarhaut J. A. Augustin H. G. Gemmill R. M. (2007). Semaphorin SEMA3F affects multiple signaling pathways in lung cancer cells. *Cancer Res.* 67 8708–8715. 10.1158/0008-5472.CAN-06-3612 17875711

[B53] Procaccia V. Nakayama H. Shimizu A. Klagsbrun M. (2014). Gleevec/imatinib, an ABL2 kinase inhibitor, protects tumor and endothelial cells from semaphorin-induced cytoskeleton collapse and loss of cell motility. *Biochem. Biophys. Res. Commun.* 448 134–138. 10.1016/j.bbrc.2014.04.063 24759231 PMC4068341

[B54] Ramkhelawon B. Hennessy E. J. Menager M. Ray T. D. Sheedy F. J. Hutchison S. (2014). Netrin-1 promotes adipose tissue macrophage retention and insulin resistance in obesity. *Nat. Med.* 20 377–384. 10.1038/nm.3467 24584118 PMC3981930

[B55] Rao J. Zhou Z. H. Yang J. Shi Y. Xu S. L. Wang B. (2015). Semaphorin-3F suppresses the stemness of colorectal cancer cells by inactivating Rac1. *Cancer Lett.* 358 76–84. 10.1016/j.canlet.2014.12.040 25529012

[B56] Rehman M. Tamagnone L. (2013). Semaphorins in cancer: biological mechanisms and therapeutic approaches. *Semin. Cell Dev. Biol.* 24 179–189. 10.1016/j.semcdb.2012.10.005 23099250

[B57] Reyes-Mugica M. Rieger-Christ K. Ohgaki H. Ekstrand B. C. Helie M. Kleinman G. (1997). Loss of DCC expression and glioma progression. *Cancer Res.* 57 382–386. 9012460

[B58] Roche J. Boldog F. Robinson M. Robinson L. Varella-Garcia M. Swanton M. (1996). Distinct 3p21.3 deletions in lung cancer and identification of a new human semaphorin. *Oncogene* 12 1289–1297. 8649831

[B59] Roy S. Bag A. K. Singh R. K. Talmadge J. E. Batra S. K. Datta K. (2017). Multifaceted role of neuropilins in the immune system: potential targets for immunotherapy. *Front. Immunol.* 8:1228. 10.3389/fimmu.2017.01228 29067024 PMC5641316

[B60] Sancak Y. Thoreen C. C. Peterson T. R. Lindquist R. A. Kang S. A. Spooner E. (2007). PRAS40 is an insulin-regulated inhibitor of the mTORC1 protein kinase. *Mol. Cell* 25 903–915. 10.1016/j.molcel.2007.03.003 17386266

[B61] Sarbassov D. D. Ali S. M. Kim D. H. Guertin D. A. Latek R. R. Erdjument-Bromage H. (2004). Rictor, a novel binding partner of mTOR, defines a rapamycin-insensitive and raptor-independent pathway that regulates the cytoskeleton. *Curr. Biol.* 14 1296–1302. 10.1016/j.cub.2004.06.054 15268862

[B62] Saxton R. A. Sabatini D. M. (2017). mTOR signaling in growth, metabolism, and disease. *Cell* 169 361–371. 10.1016/j.cell.2017.03.035 28388417

[B63] Schellenburg S. Schulz A. Poitz D. M. Muders M. H. (2017). Role of neuropilin-2 in the immune system. *Mol. Immunol.* 90 239–244. 10.1016/j.molimm.2017.08.010 28843905

[B64] Shi Y. Liu R. Zhang S. Xia Y. Y. Yang H. J. Guo K. (2011). Neural cell adhesion molecule potentiates invasion and metastasis of melanoma cells through CAMP-dependent protein kinase and phosphatidylinositol 3-kinase pathways. *Int. J. Biochem. Cell Biol.* 43 682–690. 10.1016/j.biocel.2011.01.016 21277992

[B65] Shimizu A. Mammoto A. Italiano J. E. Jr. Pravda E. Dudley A. C. Ingber D. E. (2008). ABL2/ARG tyrosine kinase mediates SEMA3F-induced RhoA inactivation and cytoskeleton collapse in human glioma cells. *J. Biol. Chem.* 283 27230–27238. 10.1074/jbc.M804520200 18660502 PMC2555994

[B66] Shimizu A. Nakayama H. Wang P. Konig C. Akino T. Sandlund J. (2013). Netrin-1 promotes glioblastoma cell invasiveness and angiogenesis by multiple pathways including activation of RhoA, Cathepsin B, and cAMP-response Element-binding Protein. *J. Biol. Chem.* 288 2210–2222. 10.1074/jbc.M112.397398 23195957 PMC3554894

[B67] Soker S. Takashima S. Miao H. Q. Neufeld G. Klagsbrun M. (1998). Neuropilin-1 is expressed by endothelial and tumor cells as an isoform-specific receptor for vascular endothelial growth factor. *Cell* 92 735–745. 10.1016/S0092-8674(00)81402-6 9529250

[B68] Solomon B. D. Mueller C. Chae W. J. Alabanza L. M. Bynoe M. S. (2011). Neuropilin-1 attenuates autoreactivity in experimental autoimmune encephalomyelitis. *Proc. Natl. Acad. Sci. U.S.A.* 108 2040–2045. 10.1073/pnas.1008721108 21245328 PMC3033275

[B69] Tadagavadi R. K. Wang W. Ramesh G. (2010). Netrin-1 regulates Th1/Th2/Th17 cytokine production and inflammation through UNC5B receptor and protects kidney against ischemia-reperfusion injury. *J. Immunol.* 185 3750–3758. 10.4049/jimmunol.1000435 20693423

[B70] Takegahara N. Takamatsu H. Toyofuku T. Tsujimura T. Okuno T. Yukawa K. (2006). Plexin-A1 and its interaction with DAP12 in immune responses and bone homeostasis. *Nat. Cell Biol.* 8 615–622. 10.1038/ncb1416 16715077

[B71] van der Zwaag B. Hellemons A. J. Leenders W. P. Burbach J. P. Brunner H. G. Padberg G. W. (2002). PLEXIN-D1, a novel plexin family member, is expressed in vascular endothelium and the central nervous system during mouse embryogenesis. *Dev. Dyn.* 225 336–343. 10.1002/dvdy.10159 12412018

[B72] van Gils J. M. Derby M. C. Fernandes L. R. Ramkhelawon B. Ray T. D. Rayner K. J. (2012). The neuroimmune guidance cue netrin-1 promotes atherosclerosis by inhibiting the emigration of macrophages from plaques. *Nat. Immunol.* 13 136–143. 10.1038/ni.2205 22231519 PMC3262880

[B73] Weiss J. M. Bilate A. M. Gobert M. Ding Y. Curotto de Lafaille M. A. Parkhurst C. N. (2012). Neuropilin 1 is expressed on thymus-derived natural regulatory T cells, but not mucosa-generated induced Foxp3^+^ T reg cells. *J. Exp. Med.* 209 1723–1742, S1. 10.1084/jem.20120914 22966001 PMC3457733

[B74] Wilson B. D. Ii M. Park K. W. Suli A. Sorensen L. K. Larrieu-Lahargue F. (2006). Netrins promote developmental and therapeutic angiogenesis. *Science* 313 640–644. 10.1126/science.1124704 16809490 PMC2577078

[B75] Wong A. W. Brickey W. J. Taxman D. J. van Deventer H. W. Reed W. Gao J. X. (2003). CIITA-regulated plexin-A1 affects T-cell-dendritic cell interactions. *Nat. Immunol.* 4 891–898. 10.1038/ni960 12910265

[B76] Wu F. Zhou Q. Yang J. Duan G. J. Ou J. J. Zhang R. (2011). Endogenous axon guiding chemorepulsant semaphorin-3F inhibits the growth and metastasis of colorectal carcinoma. *Clin. Cancer Res.* 17 2702–2711. 10.1158/1078-0432.CCR-10-0839 21349996

[B77] Xiang R. H. Hensel C. H. Garcia D. K. Carlson H. C. Kok K. Daly M. C. (1996). Isolation of the human semaphorin III/F gene (SEMA3F) at chromosome 3p21 a region deleted in lung cancer. *Genomics* 32 39–48. 10.1006/geno.1996.0074 8786119

[B78] Yadav M. Louvet C. Davini D. Gardner J. M. Martinez-Llordella M. Bailey-Bucktrout S. (2012). Neuropilin-1 distinguishes natural and inducible regulatory T cells among regulatory T cell subsets in vivo. *J. Exp. Med.* 209 1713–1722, S1–S19. 10.1084/jem.20120822 22966003 PMC3457729

[B79] Yang Q. Inoki K. Ikenoue T. Guan K. L. (2006). Identification of Sin1 as an essential TORC2 component required for complex formation and kinase activity. *Genes Dev.* 20 2820–2832. 10.1101/gad.1461206 17043309 PMC1619946

[B80] Yin K. Wang L. Zhang X. He Z. Xia Y. Xu J. (2017). Netrin-1 promotes gastric cancer cell proliferation and invasion via the receptor neogenin through PI3K/AKT signaling pathway. *Oncotarget* 8 51177–51189. 10.18632/oncotarget.17750 28881639 PMC5584240

[B81] Zaki M. H. Vogel P. Body-Malapel M. Lamkanfi M. Kanneganti T. D. (2010). IL-18 production downstream of the Nlrp3 inflammasome confers protection against colorectal tumor formation. *J. Immunol.* 185 4912–4920. 10.4049/jimmunol.1002046 20855874 PMC3104023

[B82] Zeltzer P. M. Boyett J. M. Finlay J. L. Albright A. L. Rorke L. B. Milstein J. M. (1999). Metastasis stage, adjuvant treatment, and residual tumor are prognostic factors for medulloblastoma in children: conclusions from the Children’s Cancer Group 921 randomized phase III study. *J. Clin. Oncol.* 17 832–845. 10.1200/JCO.1999.17.3.832 10071274

[B83] Zhang H. H. Lipovsky A. I. Dibble C. C. Sahin M. Manning B. D. (2006). S6K1 regulates GSK3 under conditions of mTOR-dependent feedback inhibition of Akt. *Mol. Cell* 24 185–197. 10.1016/j.molcel.2006.09.019 17052453 PMC1880887

[B84] Zygmunt T. Gay C. M. Blondelle J. Singh M. K. Flaherty K. M. Means P. C. (2011). Semaphorin-PlexinD1 signaling limits angiogenic potential via the VEGF decoy receptor sFlt1. *Dev. Cell* 21 301–314. 10.1016/j.devcel.2011.06.033 21802375 PMC3156278

